# Study on the relationship between the timing of conversion from external fixation to internal fixation and infection in the treatment of open fractures of extremities

**DOI:** 10.1186/s13018-021-02814-7

**Published:** 2021-11-07

**Authors:** Zelin Ye, Shanwen Zhao, Canjun Zeng, Ziheng Luo, Song Yuan, Runguang Li

**Affiliations:** 1 Division of Orthopaedics and Traumatology, Department of Orthopaedics, Nanfang Hospital, Southern Medical University, Guangzhou, 510515 China; 2 Department of Foot and Ankle Surgery, Center for Orthopaedic Surgery, The Third Affiliated Hospital of Southern Medical University, Guangzhou, 510610 China; 3 Orthopaedic Hospital of Guangdong Province, Guangzhou, 510610 China; 4 Academy of Orthopaedics of Guangdong Province, Guangzhou, 510610 China; 5 Guangdong Provincial Key Laboratory of Bone and Joint Degenerative Diseases, Guangzhou, 510280 China; 6 Department of Joint and Orthopedic Surgery, Nanfang Hospital, Southern Medical Univeisity, Guangzhou, 510280 China; 7 Department of Orthopedics, Linzhi People’s Hospital, Linzhi, 860000 China

**Keywords:** External fixator treatment, Open fracture, Timing of internal fixation, Gustilo classification, Bone infection

## Abstract

**Objective:**

To investigate the relationship between the infection rate and the timing of replacement of temporary external fixators with internal fixation, and the timing of immediate or delayed internal fixation after removal of temporary external fixation in the staging treatment modality of open fractures of extremities.

**Methods:**

A retrospective analysis was performed on 122 cases of open fractures of extremities. External fixators were applied at the early stage and replaced with internal fixation when the condition of soft tissues improved and inflammatory indexes dropped to the normal range or showed a steady downward trend. Depending on the carrying time of external fixators after wound closure or healing, the patients were divided into three groups; the carrying time of groups A, B, and C was ≤ 14 days, 15–28 days, and > 28 days, respectively. Depending on the immediate or delayed internal fixation after removal of external fixator, patients were divided into group a (immediate internal fixation after removal of external fixator) and group b (delayed internal fixation after removal of external fixator, 5–7 days later).

**Results:**

The infection rates of groups A, B, and C were 6.5%, 5.9%, and 23.3%, respectively. The differences among the three groups were statistically significant (*P* < 0.05). The infection rates of different Gustilo–Anderson fractures were as follows: no cases of infection out of 10 cases with type I fracture (0%); two cases of infection out of 35 cases with type II fracture (5.7%); three cases of infection out of 36 cases with IIIa fracture (8.3%); five cases of infection out of 28 cases with IIIB fracture (17.9%); and five cases of infection out of 13 cases with IIIC fracture (38.5%). The differences among the five groups were statistically significant.

**Conclusions:**

The occurrence of infection of open fractures of extremities is associated with the fracture severity (Gustilo classification). For open fractures of Gustilo types I and II, the final internal fixation should be placed as soon as possible when the recovery of general and local conditions is good and the infection is controlled.

## Introduction

With the rapid development of social economy, open fractures of limbs caused by various trauma factors become increasingly common. Open fractures of extremities are usually the result of high-energy trauma, such as traffic accidents, falling accidents, and industrial accidents, so bone and soft tissue can be severely traumatized. Some patients also have extensive skin and soft tissue defects, accompanied by exposure, injury, or defect of muscles, tendons, bones, joints, vessels, and nerves. Their treatment is characterized by long cycles and great difficulties. Once infection occurs, repeated debridement is often required, which imposes heavy economic burden on individuals, families, and countries [1]. Severe soft tissue injury and wound contamination are important factors affecting the prognosis of open fractures. If secondary infection and necrosis of bone and soft tissue occur, treatment becomes more difficult [2–5]. Injuries of patients with open fractures are complex and changeable; therefore, proper treatment procedures are needed to ensure good prognosis.

Despite the rapid development of modern medicine, the postoperative infection rate of open fractures remains high, and the postoperative limb function is poor, which seriously affects the quality of life. The methods to reduce the postoperative infection of open fractures have always been the focus of controversy.There are many factors influencing postoperative infection in open fractures, such as smoking history, diabetes mellitus, Gustilo typing,,duration of external fixator carrying time, etc. [1]. Reuss et al. [6] and Chua et al. [7] reported that there was a positive correlation between Gustilo–Anderson type and the rate of postoperative infection in open fractures. The higher the classification of the fracture, the higher the risk of infection. At present, as for the treatment of open fractures of extremities, the concept of combined treatment of fractures and soft tissue injury has been accepted by many scholars [3]. Nambi et al. [8] and O’Brien et al. [9] reported that internal fixation immediately after debridement is a safe method for Gustilo I or II fractures and some of IIIa/IIIb fractures. However, for open injuries with severe bone and soft tissue defects, many scholars have adopted the concept of orthopedic damage control for staging treatment, i.e., after emergency debridement, an external fixator is initially used to quickly fix the fracture. When the condition of the local soft tissue improves, the external fixator is removed and replaced with the deterministic internal fixation. In the initial treatment of complex fractures, external fixation is simple, convenient, and safe [3, 10, 11]. It has the advantages of rapid fixation of fractures, restoration of limb length, and avoidance of further injury of soft tissue [10, 11]. In contrast, the late treatment with an external fixator has the disadvantages of unstable fixation, easy loosening, connection between nail channels and the outside, and possible aggravation of soft tissue injury; it is also often accompanied by deep infection, nonunion, high malunion rate, and joint dysfunction [10–12]. Therefore, several studies have proved that the planned conversion from temporary external fixation to definite internal fixation at phase II is safe and avoids the inherent disadvantages of external fixators [12–14]. However, there are still some controversies on the timing and method of replacing external fixators with internal fixation at phase II; the primary dilemma is whether to apply internal fixation immediately after removal of external fixator, or to postpone the replacement with internal fixation devices [15, 16].

Postoperative infection after placing internal fixation is an important factor affecting the therapeutic effect. It is important to clarify how to reduce the incidence of postoperative infection and improve the therapeutic safety. To this end, we retrospectively analyzed 122 cases of open fractures of extremities admitted to two orthopedic centers from January 2017 to December 2019; analyzing the treatment of open fractures of extremities by sequential external fixator–internal fixation, we evaluated the relationship between postoperative infection and timing of external fixator replacement with internal fixation, and related factors, aiming to provide a reference for clinical treatment of open fractures.

## Materials and methods

### Criteria for inclusion and exclusion of cases

Case inclusion criteria were as follows: (1) age ≥ 15 years; (2) open fractures of extremities; (3) temporary external fixation of the fracture at phase I, replaced by internal fixation at phase II; (4) postoperative follow-up time ≥ 12 months; (5) complete clinical and imaging data available. Exclusion criteria were as follows: (1) patients’ choice of external fixators as the final treatment; (2) internal fixation at phase II after bone transport or bone lengthening surgery; (3) other infection foci in affected limbs during follow-up; (4) treatment with glucocorticoids or immunosuppressants for other diseases; (5) amputation due to limb necrosis caused by vascular injury during follow-up.

### General information

Based on the above eligibility criteria, 122 cases were selected, including 82 males and 27 females. Among them, 13 patients had double fractures that met the inclusion criteria. The age range was 15–70 years, with an average of 40.8 years. Causes of injuries included traffic accidents (76 cases), high falls (20 cases), machine accidents (10 cases), crushing by heavy objects (9 cases), and other causes (7 cases). Considering Gustilo classification, there were 10 cases of type I, 35 cases of type II, 36 cases of type IIIa, 28 cases of type IIIb, and 13 cases of type IIIc fractures. Multiple fractures or multiple injuries occurred in 73 cases.

### Surgery

All of the patients were treated with antibiotics and tetanus immunoglobulin soon after admission. The emergency surgery was performed using the orthopedic damage control concept. According to our experience, thorough debridement was done on open wounds at the early stage to remove contamination sources, foreign bodies, and ischemic inactivated tissues, but the remaining periosteal blood supply was retained as much as possible. Fracture reduction, correction of fracture shortening, rotation, and displacement, maintenance of fracture alignment, and installation of single-arm or combined external fixators were performed under fluoroscopy guidance. The fracture site stability was tested during the operation. If it was stable, cross-joint fixation was unnecessary. If not, cross-joint fixation was applied to increase stability. Limited internal fixation by Kirschner wire was applied depending on the fracture characteristics if necessary.

Different treatments were selected depending on the wound conditions: (1) direct suture of tension-free wound; (2) coverage with vacuum sealing drainage (VSD) or KCI sponge at phase I, 4–7 days each time, for severe tension wounds or skin defects. According to the wound situation, patients with severe contamination, unclear boundaries of tissue necrosis, or wound infection received repeated debridement as needed. If the surrounding granulation tissue status was good, flap transposition, flap dissociation, and free skin graft were performed at phase II.

When soft tissue swelling completely subsided, skin wrinkled, there were no local infection symptoms and signs, such as swelling and obvious inflammatory secretions in the wound, the general condition improved, and inflammatory indexes, such as white blood cells (WBCs), C-reactive protein (CRP), erythrocyte sedimentation rate (ESR), and procalcitonin (pro-CT), decreased steadily or decreased to the normal range [3, 10]. Patients were divided into the immediate group and delayed group (5–7 days) according to the nail path. Patients in the immediate group replaced the internal fixator immediately after removing the temporary external fixator. After removing the temporary external fixator, the patients in the delayed group fixed the affected limb with plaster and then replaced the internal fixator when the nail path was healed (5–7 days). To replace the internal fixation, we used small incision reduction or closed reduction and implanted steel plates or intramedullary nails for fixation.

### Definition of carrying time of external fixators and infection

Carrying time of external fixators was measured from suture or repair time of open fracture wounds to internal fixation. Infection after internal fixation was defined as bone tissue infection with or without surrounding soft tissue infection after the replacement of external fixator with internal fixation due to pathogenic microbial contamination or low immunity of patients. For diagnostic criteria of bone infection, we referred to the consensus formulated by the International Association for Internal Fixation Research in 2017 [17]. In this study, the infection was classified into three grades based on the infection degree and the treatment method: Grade 1, mild infection, which could be improved by conservative treatment; Grade 2, moderate infection, needing debridement surgery; Grade 3, severe infection, where internal fixation had to be removed, followed by thorough debridement, bone transport, or fibula transplantation after lesion clearance. Infections of varying degrees may occur during follow-up, and we included the patients with the highest level of infection in this study.

### Methods

According to the carrying time of external fixators after wound closure or healing, the patients were divided into three groups (A, B, and C); the carrying time in groups A, B, and C was ≤ 14 days, 15–28 days, and > 28 days, respectively. Depending on immediate or delayed replacement with internal fixation, the cases were divided into two groups (a and b). In group a, internal fixation was placed immediately after removal of external fixators. In group b, internal fixation was delayed 5–7 days after removal of external fixators. The medical records of the patients were collected, and the corresponding imaging results and results of WBCs, CRP, ESR, and other inflammatory indices were collected simultaneously. The incidence of deep tissue infection (osteomyelitis) and efficacy were compared between the groups. Chi-square test or Fisher exact probability test was used to compare the incidence of infection after internal fixation placement between the groups.

## Results

In this study, 109 patients and 122 affected limbs were included. No statistically significant intergroup differences were found in the general preoperative characteristics (*P* > 0.05), which indicated the comparability of the groups (Table 1). There were 15 cases of infection after replacement of external fixator with the final internal fixation, and the total infection rate was 12.3%. Among them, there were four cases of mild infection, six cases of moderate infection, and five cases of severe infection. After a series of treatments, 14 patients with infection were cured, whereas one patient eventually developed chronic osteomyelitis and received amputation (Table 2).

**Table 1:** Patient characteristics

Demographic variables	Data
Number of patients	109
Number of limbs	122
Age (mean ± SD, years)	40.8 ± 13.7
Gender (Males, %)	82 (75.2%)
Smokers	33 (30.3%)
*Mechanism of injury*	
Traffic accident	76 (62.3%)
Fall	20 (16.4%)
Machine injury	10 (8.2%)
Bruised	9 (7.4%)
Others	7 (5.7%)
*Temporary EF duration group*	
≤ 14 days (%)	62 (50.8%)
15–28 days (%)	17 (13.9%)
> 28 days (%)	43 (35.2%)
Conversion from EF to IF (Directly, %)	92 (75.4%)
*Definite IF*	
Locking plate	89 (73%)
Intramedullary nail	33 (27%)

**Table 2:** Demographic characteristics of the patients with fracture

Fracture	Data
Gustilo classification	122
I (%)	10 (8.2%)
II (%)	35 (28.7%)
IIIA (%)	36 (29.5%)
IIIB (%)	28 (23.0%)
IIIC (%)	13 (10.7%)
*Outcome*	
Healed (%)	107 (87.7%)
Infected (%)	15 (12.3%)
*Infection degree*	
Mild (%)	4 (26.7%)
Moderate (%)	6 (40.0%)
Severe (%)	5 (33.3%)

The infection rates of different Gustilo–Anderson injuries were compared. The infection rate of type I was 0% (0/10), type II—5.7% (2/35), type IIIa—8.3% (3/36), type IIIb—17.9% (5/28), and type IIIC—38.5% (5/13). By comparing the postoperative infection rates of different types of Gustilo fractures after internal fixation placement, it was found that the postoperative infection rate increased with the increase in the severity of open fractures. The differences were statistically significant (Table 3). The postoperative infection rate of the immediate replacement with internal fixation after the external fixation was removed was 10.5%, while the postoperative infection rate of the immediate replacement with internal fixation after the external fixation was removed was 18.5%; the difference between two groups was not statistically significant.

**Table 3:** Comparison of infection rates among Gustilo fracture types

Group	Infection					*P* value
	Yes	No	Overall	Ratio (%)	X^2^/Fisher	
I	0	10	10	0	11.370	0.023
II	2	33	35	5.7%		
IIIA	3	33	36	8.3%		
IIIB	5	23	28	17.9%		
IIIC	5	8	13	38.5%		
Overall	15	107	122	12.3%		

In the group where the carrying time of external fixator was ≤ 14 days, the infection rate was 6.5% (4/62 cases). The infection rates were 5.9% (1/17 cases) and 23.3% (10/43 cases) in the group with the carrying time of 15–28 days and that of > 28 days, respectively. With the extension of the carrying time of temporary external fixators, the postoperative infection rates of the three groups showed an overall increasing trend, and the differences among three groups were statistically significant, supporting the principle of replacing external fixators with final internal fixation for open fracture patients as soon as possible. We found a 6.5% infection rate (4/62 cases) in patients in whom external fixators had been removed within 14 days. The infection rate after 14 days was 18.3% (11/60). The comparison of the infection rates after internal fixation between the two groups showed a significant difference. In addition, the infection rate in patients in whom external fixators had been removed within 28 days was 6.3% (5/79); the infection rate after 28 days was 23.3% (10/43 cases), which was significantly different (Tables 4, 5, 6).

**Table 4:** Comparison of postoperative infection based on the carrying time of temporary external fixators

Group	Infection					*P* value
	Yes	No	Overall	Ratio (%)	X^2^/Fisher	
≤ 14 days	4	58	62	6.5	7.043	0.030
15–28 days	1	16	17	5.9		
> 28 days	10	33	43	23.3		
Overall	15	107	122	12.3

**Table 5:** Comparison of postoperative infection between the 2 groups with temporary external fixation carried for ≤ 14 days versus > 14 days

Group	Infection					*P *value
	Yes	No	Overall	Ratio (%)	X^2^/Fisher	
≤ 14d	4	58	62	6.5	3.992	0.046
> 14d	11	49	60	18.3		
Overall	15	107	122	12.3

**Table 6:** Comparison of postoperative infection between the 2 groups with temporary external fixation carried for ≤ 28 days versus > 28 days

Group	Infection					*P* value
	Yes	No	Overall	Ratio (%)	X^2^/Fisher	
≤ 28d	5	74	79	6.3	7.398	0.007
> 28d	10	33	43	23.3		
Overall	15	107	122	12.3		

In addition, comparing the postoperative infection rate in the group of immediate replacement of internal fixation after removal of temporary external fixation and in the group of replacement of internal fixation after 5–7 days extension after removal of temporary external fixation, we found that the infection rate was 10.5% (10/95) in the immediate group and 18.5% (5/27) in the interval group, while there was no statistical difference in the comparison between the two groups (Table 7).

**Table 7:** Comparison of postoperative infection between the immediate group and the 5–7 days interval group with internal fixation replacement

Group	Infection					*P* value
	Yes	No	Overall	Ratio (%)	X^2^/Fisher	
Immediate	10	85	95	10.5	0.615	0.433
interval	5	22	27	18.5		
Overall	15	107	122	12.3		

**Fig. 1:**
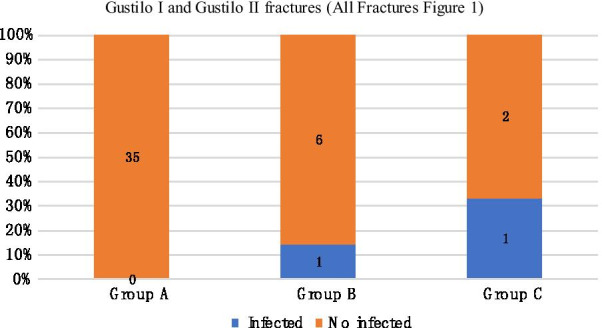
Gustilo I and Gustilo II fractures.The proportion of infected patients in Group A, Group B, and Group C (duration from EF to IF ≤ 14 days, 15–28 days, > 28 days, respectively) with Gustilo I and Gustilo II fractures. *P* < 0.05

**Fig. 2:**
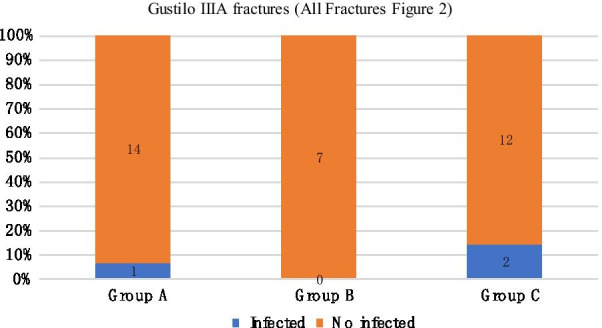
Gustilo IIIA fractures.The proportion of infected patients in Group A, Group B, and Group C (duration from EF to IF ≤ 14 days, 15–28 days, > 28 days, respectively) with Gustilo IIIA fractures. *P* > 0.05

**Fig. 3:**
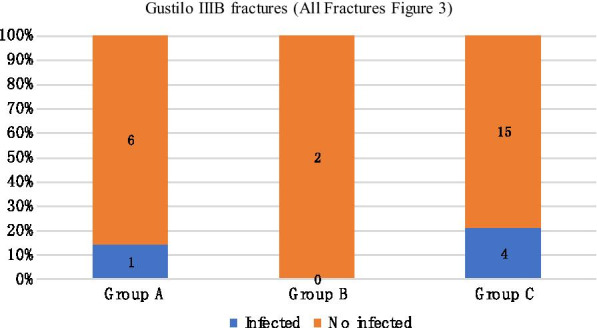
Gustilo IIIB fractures.The proportion of infected patients in Group A, Group B, and Group C (duration from EF to IF ≤ 14 days, 15–28 days, > 28 days, respectively) with Gustilo IIIB fractures. *P* > 0.05

**Fig. 4:**
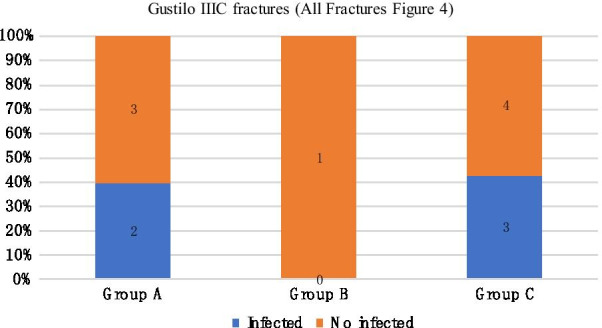
Gustilo IIIC fractures.The proportion of infected patients in Group A, Group B, and Group C (duration from EF to IF ≤ 14 days, 15–28 days, > 28 days, respectively) with Gustilo IIIC fractures. *P* > 0.05

We compared the infection rates of different temporary external fixator indwelling time under the same Gustilo classification. In mild injury (Gustilo I and II), the infection rate of group A was 0%, that of group B was 14.3%, and that of group C was 33.3%. The differences among the three groups were statistically significant (Fig. 1). In Gustilo type IIIa, the infection rates of groups A, B, and C were 6.7%, 0%, and 14.3%, respectively (Fig. 2). In Gustilo type IIIb, the infection rates of groups A, B, and C were 14.3%, 0%, and 21.1%, respectively (Fig. 3). In Gustilo IIIc type, the infection rates of groups A, B, and C were 40%, 0%, and 42.9%, respectively (Fig. 4). In the Gustilo IIIa, IIIb, and IIIc stratification, the differences among the three groups were not statistically significant (*P* > 0.05).

## Discussion

Open fractures of extremities with severe soft tissue injury are mostly caused by high-energy injury. There are no effective solutions for the infection risk caused by latent bacteria in deep tissues, and the risk of deep tissue infection (osteomyelitis) is high after operations. The combined treatment of fracture and soft tissue injury can ensure better prognoses. Early debridement, soft tissue coverage, and early fracture fixation are particularly important [18]. However, patients receiving early internal fixation of limb open fractures with severe soft tissue injury have a high incidence of infections, which seriously hampers the therapeutic effect [19, 20]. External fixation braces play a very important role in the treatment of open fractures of the extremities [1]. In addition to the role of temporary fixation, the external fixator can also be used as a terminal fixation to treat open fractures of the limbs. The external fixator has a small soft tissue trauma effect, which limits the impact on bone blood supply [21, 22]. Inan et al. [23] compared the efficacy of Ilizarov external fixator (IEF) and unreamed tibial nailing (UTN) and found that the healing time of IEF (19 weeks) was shorter than that of UTN (21 weeks). However, external fixation alone, especially unstable fractures, may be complicated by malunion, loss of reduction, refracture, and needle tract infection. According to reports, the incidence of these complications is as high as 55% of malunion, 23% of loss of reduction and 21% of refracture [24–26]. In addition, carrying the external fixator for a long time is very inconvenient life for the patient, and some studies have found that the patient even has mental illness [27, 28]. Therefore, more and more patients believe that as soon as possible, the external fixator should be replaced with internal fixation [12–14]. In addition, considering that open fractures caused by high-energy trauma are usually accompanied by multiple injuries, and systemic conditions of the patients are poor, it is difficult for them to tolerate early internal fixation [29, 30]. Currently, for severe open fractures, the damage control orthopedics (DCO) theory has been accepted by most clinicians [31]. Blachut et al. [32] reported the treatment of open tibial fractures with planned temporary external fixators, followed by internal fixation, and achieved satisfactory results. In recent years, a number of studies have proven the safety and reliability of this therapeutic modality, which also conforms to the DCO concept, which has resulted in its acceptance by an increasing number of clinicians [3, 33, 34]. In this study, 109 patients (122 affected limbs) were treated using this approach. The incidence of infections in our study was 12.3%, which is consistent with that in previous reports. Therefore, the planned temporary external fixation followed by internal fixation is safe and effective for treating open fractures of extremities.

Despite the data that planned conversion therapy is safe for severe open fractures of extremities, the best timing for internal fixation has not yet been thoroughly explored. Bhandari et al. [27] conducted a meta-analysis of open fractures of tibial shaft. They found that the infection rate increased when the temporary external fixator indwelling time was > 28 days and the conversion interval at phase II was > 14 days; therefore, they suggested that external fixator indwelling time should not exceed 28 days and the conversion interval at phase II should not exceed 14 days. Some scholars believe that temporary external fixator can be switched to deterministic internal fixation when patients’ general conditions or local soft tissue improves after 5–10 days of indwelling [16, 35, 36]. Other scholars suggest that an indwelling duration of 5–14 days for temporary external fixators is relatively safe [37–39]. In this study, to exclude the effect of healing time of open fracture wounds on the time for replacing external fixators with internal fixation, the carrying time of external fixators was specifically defined as the carrying time of external fixators after wound closure or repair. We found that for Gustilo type I and II injuries, the infection rate significantly increased with the increase of the carrying time of temporary external fixators. In other words, for mild open fractures of extremities, longer carrying time of temporary external fixators is associated with a higher overall risk of infection.

It is also controversial whether to apply internal fixation immediately after removal of temporary external fixators. Some scholars believe that when a temporary external fixator is retained for more than 14 days, after removing the temporary external fixator, the final internal fixation should be replaced only after the healing of nail path and after the inflammatory indices return to normal (usually 5–7 days) [28, 33]. Nowotarski et al. [39] compared the infection rates of replacement with internal fixation at phases I and II, and suggested that temporary external fixators can be directly replaced by internal fixation within 1–2 weeks. In our study, by comparing the infection rates of replacement with internal fixation at phases I and II, we found that there was no significant difference between the two, which is also consistent with previous reports [33, 40, 41].

It has been reported [7, 42] that the Gustilo–Anderson type is closely related to the occurrence of open fracture infection. Higher types are associated with a higher infection rate and fracture nonunion rate. It was reported [43] that the infection rates of type I, II, IIIA, IIIB, and IIIC open fractures were 2%, 2–10%, 5–10%, 10–50%, and 25–50%, respectively. In our study, the infection rates of type I, II, IIIA, IIIB, and IIIC open fractures were 0%, 5.7%, 8.3%, 17.9%, and 38.5%, respectively, and the overall infection rate was 12.3%. Our results are consistent with those reported in previous literature. We also found that differences among the five groups were statistically significant, indicating that the infection rate of limb open fractures after sequential temporary external fixation and internal fixation was related to the degree of limb open injury. Due to the different fracture severities, the severities of soft tissue injury also differ. The differences in soft tissue injury are mainly manifested in wound size, skin and muscle injury, vascular injury, bone tissue injury, and contamination degree. These may also lead to differences in postoperative infection rates [1]. Lua et al. [44] compared the infection rates of open fractures with different injury degrees and found that the incidence of infection-related complications in patients with Gustilo III tibial open fractures was 3.72 times higher than that in patients with Gustilo I/II type. They suggested that higher degree of open injury is associated with a greater damage of soft tissue and more serious contamination, which may lead to unclear boundaries of soft tissue necrosis, difficult debridement, and increased infection rate.

This study also has the following limitations: (1) It was not randomized, but retrospective study, so there was potential bias in the results. (2) The age of doctors on duty for emergency debridement after admission was uneven, and the operations were performed by doctors at different hospitals, which may have affected the results. (3) This study included Gustilo I and II open fractures. External fixation may be the preferred treatment for Gustilo–Anderson III fractures, and IF may be the preferred treatment for most type I open fractures. Therefore, it is questionable whether the Gustilo–Anderson I/II open fractures with injuries or fractures and Gustilo–Anderson III open fractures should be analyzed together. 4) There is a close correlation between indwelling time of temporary external fixator and injury degree, and the injury degree is an important factor in postoperative infection. In this study, the sample size of Gustilo III was insufficient, which prevented better comparison of the infection rates between different temporary external fixator indwelling times in Gustilo type IIIa and above. Therefore, we intend to further explore the relationship between the carrying time of temporary external fixators and infection in open fractures of Gustilo type IIIa and above.

However, apart from these limitations, this study conducted in two orthopedic centers clarified that sequential external fixator–internal fixation staging treatment was safe and effective for open fractures of extremities. The main factor affecting postoperative infection of open fractures of extremities was the degree of limb injury, and the infection rate increased with increasing the degree of the injury. The indwelling time of temporary external fixators had a certain effect on the postoperative infection rate of mild open fractures; longer indwelling time of temporary external fixation was associated with a higher risk of postoperative infection. For open fractures of Gustilo IIIa and above, additional clinical investigation is needed to clarify whether the duration of temporary external fixator indwelling affects the postoperative infection rate.

In summary, there are some difficulties in the treatment of open fractures of extremities. For open fractures with mild injury, the replacement of external fixator with internal fixation as early as possible on the basis of good infection control, soft tissue, and general conditions is conducive to reducing the risk of infection. Internal fixation can be applied immediately or delayed after removal of the external fixator, but the decision should be made based on the infection indices and the reaction of the nail path.

## Data Availability

None.

## Abbreviations

WBC: White blood cells
CRP: C-reaction protein
ESR: Erythrocyte sedimentation rate
pro-CT: Procalcitonin
